# Comparison of SNCG and NEFH Promoter–Driven Expression of Human SIRT1 Expression in a Mouse Model of Glaucoma

**DOI:** 10.1167/tvst.13.8.37

**Published:** 2024-08-23

**Authors:** Nuala O'Neill, Miranda Meng, Brahim Chaqour, Kimberly Dine, Neha Sarabu, Jennifer C. Pham, Kenneth S. Shindler, Ahmara G. Ross

**Affiliations:** 1 Department of Ophthalmology, University of Pennsylvania, Philadelphia, PA, USA; 2 F.M. Kirby Center for Molecular Ophthalmology, Scheie Eye Institute, University of Pennsylvania Perelman School of Medicine, Philadelphia, PA, USA; 3 Department of Neurology, University of Pennsylvania, Philadelphia, PA, USA

**Keywords:** ATOH7, NEFH, SNCG, SIRT1, glaucoma

## Abstract

**Purpose:**

Adeno-associated virus (AAV) demonstrates promise in delivering therapeutic genes to retinal ganglion cells (RGCs). Delivery of neuroprotective genes is constrained by packaging size and/or cell selectivity. This study compares the ability of the RGC-selective gamma-synuclein (SNCG) promoter and the smaller RGC-selective neurofilament heavy chain (NEFH) promoter, as well as portions of the RGC-selective atonal bHLH transcription factor 7 (ATOH7) enhancer, to drive gene expression in RGCs.

**Methods:**

AAV2 constructs with green fluorescent protein (GFP) or human sirtuin 1 (hSIRT1) driven by cytomegalovirus (CMV) enhancer and NEFH promoter (AAV2–eCMV–NEFH) or distal active sequences of the ATOH7 enhancer (DiATOH7) with the SNCG promoter (AAV2–DiATOH7–SNCG) were intravitreally injected into C57BL/6J mice. RGCs were immunolabeled with Brn3a antibodies and counted. AAV constructs with the utmost transduction efficiency were used to test the therapeutic efficacy of the hSIRT1 gene in 12-week-old C57BL/6J mice subjected to microbead (MB)-induced intraocular pressure (IOP) elevation. Visual function was measured using optokinetic responses (OKRs).

**Results:**

The eGFP transduction efficiency of AAV2–eCMV–NEFH was similar to that of AAV2–eCMV–SNCG and AAV2–DiATOH7–SNCG. When combined with the SNCG promoter, a larger ATOH7 enhancer was less efficient than the shorter DiATOH7 enhancer. Similarly, the hSIRT1 efficiency of AAV2–eCMV–NEFH was similar to that of AAV2–eCMV–SNCG. The latter two vectors were equally efficient in increasing RGC survival and improving visual function in the mouse model of MB-induced IOP elevation.

**Conclusions:**

SNCG and NEFH promoters represent two equally efficient and comparable RGC selective promoter sequences; however, the NEFH promoter offers a smaller packaging size.

**Translational Relevance:**

Smaller enhancer–promoter combinations can be used to deliver larger genes in human cells and for treatment in optic neuropathies including glaucoma.

## Introduction

The development of gene therapy continues to hold promise in revolutionizing the clinical management of eye disease. In this regard, the widespread use and growing popularity of adeno-associated virus (AAV) presents a significant opportunity for effectively delivering essential therapeutic genes to address retinal diseases.^1^ Today, over 70 phase I and II clinical trials have been conducted to explore the potential of AAV-mediated therapies in ocular conditions; however, despite these efforts, many trials have yet to replicate the preclinical and subsequent clinical success observed with voretigene neparvovec-rzyl in treating Leber's congenital amaurosis type 2.^1^^–^^3^

Reflecting on these “failures,” the translational gap has revealed distinct obstacles hindering the broader adoption of these therapies for ocular diseases.^4^^,^^5^ The need for a lower therapeutic dose and more effective viral vectors to deliver therapeutic payloads to specific cell types is becoming increasingly important.^6^ Ocular therapeutics are no exception to this requirement. Extensive research in AAV molecular biology has identified crucial factors for optimizing transduction efficiency, including AAV serotype, delivery method, vector preparation, and promoter type.^7^ One experimental approach to optimizing the utilization of AAV-mediated therapies, particularly in retinal ganglion cells (RGCs), involves addressing inefficiencies in the promoters and regulatory elements of the AAV vector within target cells.^7^^–^^11^ Furthermore, it is important to note that the therapeutic payload of AAV vectors is constrained by their 4.7-kb carrying capacity,^4^^,^^12^^,^^13^ underscoring the need for more emphasis on improving the strength and cell selectivity of gene expression with smaller more effective promoters.

RGCs serve as the primary conduit for visual information from the retina to the brain via the optic nerve and are essential for functional vision. RGCs are, however, vulnerable to damage and degeneration, leading to sight-threatening conditions.^14^ Glaucoma, a progressive optic neuropathy characterized by the loss of RGCs and their axons, is one of the most prevalent diseases involving RGC dysfunction.^15^ Additionally, conditions such as optic neuritis, ischemic optic neuropathy, and traumatic optic neuropathy also affect RGCs, resulting in vision impairment or loss. A promising treatment for RGC neuroprotection is silent information regulator 1 (sirtuin-1/SIRT1/hSIRT1).^8^^–^^11^ SIRT1 is a ubiquitously expressed nicotinamide adenine dinucleotide (NAD+)-dependent deacetylase that functions to prevent both oxidative stress and inflammation in various tissues. Non-specific enhancement of the pathway and upregulation of SIRT1 through protein expression attenuates RGC loss and preserves visual function in multiple models of neurodegenerative disease.^8^^–^^10^^,^^16^^,^^17^ Further probing into the SIRT1 mechanism of neuroprotection in RGCs suggests that it involves signaling through peroxisome proliferator–activated receptor gamma coactivator-1 alpha (PGC1-α) and enhancement of mitochondrial function and will continue to be investigated.^18^ Our hypothesis suggests that leveraging hSIRT1 could serve as a benchmark to refine AAV vectors for enhanced expression and therapeutic efficacy with smaller packaging sizes that can later potentially be utilized to accommodate larger therapeutic genes. This approach may facilitate the development of smaller AAV vectors capable of delivering a broader range of therapies.

Previously reported vectors optimized for RGC-selective expression include combined cytomegalovirus (CMV) enhancer (0.304 kb)–gamma-synuclein (SNCG) promoter (0.953 kb)–simian virus 40 (SV40) intron DNA fragment (0.097 kb), along with the poly(A) tail (0.208 kb) and woodchuck hepatitis virus post-transcriptional regulatory element (WPRE) (0.589 kb) sequence, totaling approximately 2.151 kb.^8^ Thus, constructs containing these elements can accommodate a therapeutic transgene of up to 2.549 kb. We aimed to identify an AAV2 combination that has equal or greater efficiency with a smaller packaging size. This study investigated the expression and potential therapeutic efficacy of three enhancer sequences, CMV and a smaller and larger portion of the atonal homology (atonal bHLH transcription factor 7 [ATOH7]) enhancer, in conjunction with RGC-selective promoters, neurofilament heavy chain (NEFH)^19^ and SNCG.^20^ Vectors were designed with a goal to maintain or improve the transduction efficiency of enhanced green fluorescent protein (eGFP) or therapeutic hSIRT1 expression at a reduced packaging size.

The ATOH7 competence factor is crucial for developing multipotent progenitors into vertebrate RGCs and shows dynamic expression patterns during retinal histogenesis.^21^ Extensive research has been conducted on the regulation of ATOH7 through select regulatory sequences upstream at potential enhancer sequences. Studies exploring the binding and activity of these regulatory elements have revealed four distinct open segments, including two conserved non-coding elements (CNEs) and active distal (Di) and proximal (Pr) sequences as potential areas of transcriptional regulation. These segments have emerged as novel duplicate sequences for enhancing the expression of target gene selectively in RGCs.^22^

Recent studies have shown that the novel 2.2-kb murine *NEFH* gene promoter preferentially and efficiently drives eGFP expression in RGCs compared to a ubiquitous CMV promoter.^19^ A smaller, more efficient, and tissue-specific promoter was identified by further analysis of a 199-bp sequence upstream of the *NEFH* gene which selectively drives eGFP expression in RGCs.

Employing AAV2 as a viral serotype, each enhancer and promoter was coupled with either eGFP or hSIRT1 to evaluate transduction efficiency, and the therapeutic potential of the most highly transduced construct was tested in a mouse ocular hypertensive disease model. This comparative analysis aimed to identify novel enhancer and promoter combinations that can contribute to developing a RGC-selective AAV vector with increased packaging capacity, potentially capable of facilitating the delivery of a broader range of therapeutic payloads.

## Materials and Methods

### Animals

Male and female C57BL/6J mice were obtained from The Jackson Laboratory (Bar Harbor, ME) and bred under a 12-hour light/dark cycle. The animals were housed at the University of Pennsylvania animal facility in accordance with the ARVO Statement for the Use of Animals in Ophthalmic and Vision Research, as well as guidelines established by the Institutional Animal Care and Use Committee and federal regulations.

### AAV Design and Preparation

The following AAV vectors were produced and packaged by the University of Pennsylvania Research Vector Core at the Center for Advanced Retinal and Ocular Therapeutics: AAV2.CMV.hSNCG.eGFP.WPRE (V1.G), AAV2.CMV.NEFH.eGFP.WPRE (V2.G), AAV2.ATOH7.hSNCG.eGFP.WPRE (V3.G), AAV2.diATOH7.hSNCG.eGFP.WPRE (V4.G), AAV2.CMV.hSNCG.hSIRT1.WPRE (V1.S), AAV2.CMV.NEFH.hSIRT1.WPRE (V2.S), and AAV2.diATOH7.hSNCG.hSIRT1.WPRE (V4.S). The original proviral vector consisted of the CMV enhancer derived from InvivoGen pDRIVE CAG plasmid (San Diego, CA), with the human SNCG promoter subcloned into the vector.^20^ The NEFH promoter, large ATOH7, and smaller diATOH7 enhancer sequences were synthesized using GeneScript (GeneScript Biotech, Piscataway, NJ) and subcloned using the construct containing the CMV enhancer and SNCG promoter. Through the use of limited restriction enzymes, enhancer and promoter sequences were replaced within the same original CMV–SNCG construct. The cDNA encoding eGFP protein, the WPRE element (i.e., nucleotides 1093–1685; GenBank accession no. J04514), and the bovine growth hormone (bGH) polyadenylation signal were also subcloned. Codon-optimized hSIRT1 (transcript variant 1) cDNA clones were obtained from OriGene (Rockville, MD). AAV expression cassettes were flanked by the AAV2 inverted terminal repeats. Sequences were amplified with DNA polymerase (New England Biolabs, Ipswich, MA) and cloned into the AAV expression plasmid using a commercial cloning kit (Clontech Laboratories, Mountain View, CA). All AAV vectors were packaged using previously described methods and purified with CsCl gradient.^23^ Vectors were stored in 0.001% Pluronic F-68 at −80°C until just before use and then diluted to the concentration indicated in the text.

### Intravitreal Injection

A cohort of 4-week-old mice were anesthetized using isoflurane inhalation. A 10-µL Hamilton syringe (701 RN; Hamilton Company, Reno, NV) attached to a 33-gauge sharp-end needle was inserted into the vitreous cavity with the needle tip placed directly above the optic nerve head. A volume of 2 µL of AAV preparation containing 3 × 10^9^ vector genomes was injected into each eye. In the therapeutic model, both eyes of each mouse received either sham or therapeutic injections, allowing each eye to serve as an independent endpoint for raised IOP or no raised IOP following microbead injection into one eye only. This also was chosen to avoid transsynaptic contamination of vectors.^24^

### Microbead-Induced Ocular Hypertension

Ocular hypertension was induced as described previously.^11^ Mice received intracameral injections of 1.5 µL of magnetic microbeads (Invitrogen Dynabeads M-450 Epoxy, cat. no. 14011, 1.6 × 10^6^ beads/µL saline; Thermo Fisher Scientific, Waltham, MA) in the right eye and concurrent injections of balanced salt solution (BSS) in the left eye. IOP was measured in each eye in the morning 3 weeks before the first injection and then weekly with the iCare TONOLAB tonometer (iCare, Vantaa, Finland) in awake, unanesthetized animals.

### Optokinetic Response Recordings

Visual function was assessed by measuring the optokinetic responses (OKRs) using commercial software and apparatus (OptoMetry; CerebralMechanics, Medicine Hat, AB, Canada) as previously described.^25^ The OKR was determined as the highest spatial frequency where mice tracked a 100% contrast grating projected at different spatial frequencies. Briefly, mice were placed unrestrained on a platform in the center of four computer monitors with a video camera above the platform to capture the movement of the mouse. A rotating cylinder with vertical sine wave grating was computed and projected to the monitors.^26^ The sine wave grating, consisting of black and white bars at 100% contrast and 12°/s, provides a virtual-reality environment to measure the spatial acuity of the left eye when it rotates clockwise and the right eye when it rotates counterclockwise. Thus, the asymmetry of the optokinetic motor reflex allows for testing of visual function for each eye separately.^27^ Testing was performed by a masked investigator, and OKRs were recorded for both eyes.

### Wholemount Immunofluorescent Staining and RGC Quantification

RGC staining and quantification were performed as previously described.^9^^,^^11^^,^^17^^,^^28^ Mice were euthanized by cervical dislocation, and their eyes were fixed in 4% paraformaldehyde solution for 1 hour at room temperature. Following extraction of the entire retina from the optic cups, tissues were then permeabilized in 0.5% Triton X-100 in phosphate-buffered saline (PBS) with freezing at –80°C for 15 minutes. Retinas were blocked in a blocking solution of 2% Triton X-100, 2% normal bovine serum albumin (cat. no. A2153-10G; Sigma-Aldrich, St. Louis, MO), and PBS for 30 minutes. Samples were incubated with a primary antibody solution containing goat anti-hSIRT1 antibody (1:1000 dilution; ProSci, Poway, CA) and/or rabbit polyclonal anti-Brn3a antibody (1:1000 dilution; Synaptic Systems, Goettingen, Germany) overnight at 4°C.^29^ Retinas were washed with PBS six times and then incubated with a secondary antibody solution of 1:500 Invitrogen Donkey anti-Rabbit IgG (H+L) Highly Cross-Adsorbed Secondary Antibody, Alexa Fluor Plus 594 (cat. no. A32754; Thermo Fisher Scientific); 1:1000 Invitrogen Donkey anti-Rabbit IgG (H+L) Highly Cross-Adsorbed Secondary Antibody, Alexa Fluor 488 (cat. no. A21206; Thermo Fisher Scientific); or Donkey anti-Goat IgG (H+L) Cross-Adsorbed Secondary Antibody, Alexa Fluor 488 (cat. no. A11055; Thermo Fisher Scientific) for 1 hour. Retinas were flattened and mounted, vitreous side upward, with Fluoromount G mounting medium (cat. no. 0100-01; SouthernBiotech, Birmingham, AL). After masking photographers to the experimental cohorts, photographs were taken using a fluorescence microscope and Nikon NIS-Elements imaging software (Nikon, Tokyo, Japan) at 40× magnification in 12 standard fields per retina: 1/6, 3/6, and 5/6 of the retinal radius from the center in each quadrant. The 12 fields covered a total area of 0.407 mm^2^/retina. Mice injected with the AAV vectors expressing the eGFP reporter were stained with anti-Brn3a antibody only for RGC colocalization with eGFP signal. The number of retinal eGFP^+^ and Brn3^+^ cells was determined by quantifying the number of colocalized red and green cells to determine transduction efficiency.

### Statistical Analyses

All data are represented as mean ± standard error of the mean (SEM). Differences between treatment groups with respect to OKR IOPs were compared using repeated-measures analysis of variance (ANOVA), and RGC staining was quantified by one-way ANOVA, followed by Tukey's honestly significant difference (HSD) test, as indicated, using Prism 9.0 (GraphPad, Boston, MA). Differences were considered statistically significant at *P* < 0.05.

## Results

### Comparative Analysis of Novel ATOH7 Enhancer and NEFH Promoter Sequence Transduction Potential in RGCs

**Figure 1.**
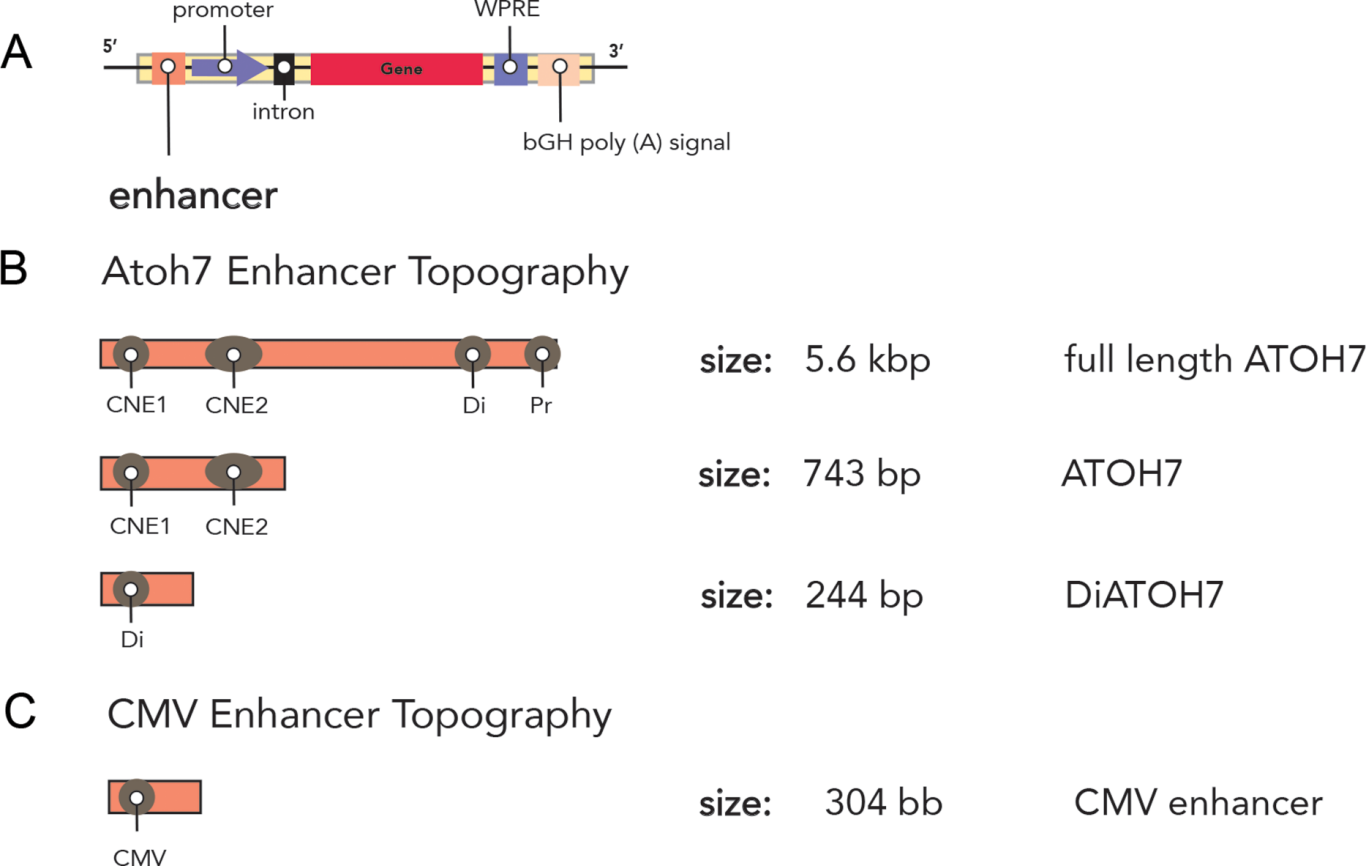
Topographic maps of experimental enhancers. (**A**) Representative vector map demonstrating the following expression cassette components: enhancer, promoter, intron, gene of interest (gene), post-transcriptional WPRE, and bovine growth hormone poly-AAA signal (bGH poly-A). (**B**) Topography of the ATOH7 enhancer sequences demonstrating the two CNEs, the Di sequence, and the Pr sequence. (**C**) Topography of the CMV enhancer sequence. Sizes of each enhancer have been included.

**Figure 2.**
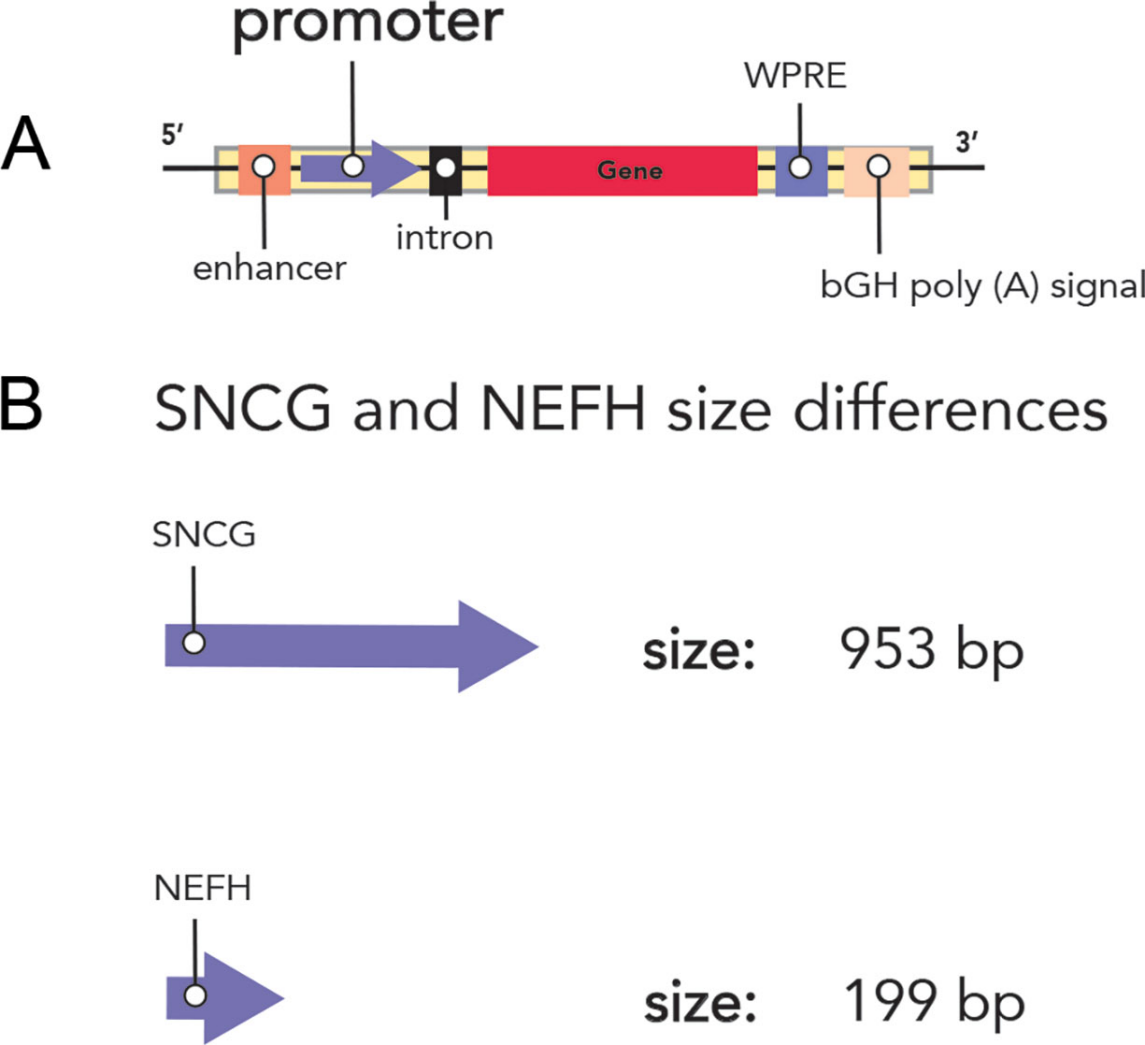
Topographic maps of experimental promoters. (**A**) Representative vector map demonstrating the following expression cassette components: enhancer, promoter, intron, gene of interest (gene), post-transcriptional WPRE, and bGH poly-A. (**B**) Topography of the SNCG and NEFH promoter sequences. Sizes of each enhancer have been included.

The enhancer element, one of the regulatory sequences used in gene delivery vectors (Fig. 1A), termed “full-length ATOH7” (Fig. 1B), spans 5.6 kbp. However, independent CNEs and Di sequences have been utilized to identify novel and smaller potential RGC-selective enhancer sequences (Fig. 1B) that are more comparable in size to the constitutive CMV enhancer. Previously, it was demonstrated that the 953-bp SNCG promoter (Fig. 2B) exhibits specific and potent sustained transgene expression in both mouse and primate RGCs,^8^^–^^11^^,^^24^ and a recent study showed that a smaller 199 NEFH promoter (Fig. 2B) also drives RGC-selective expression.^19^

**Figure 3.**
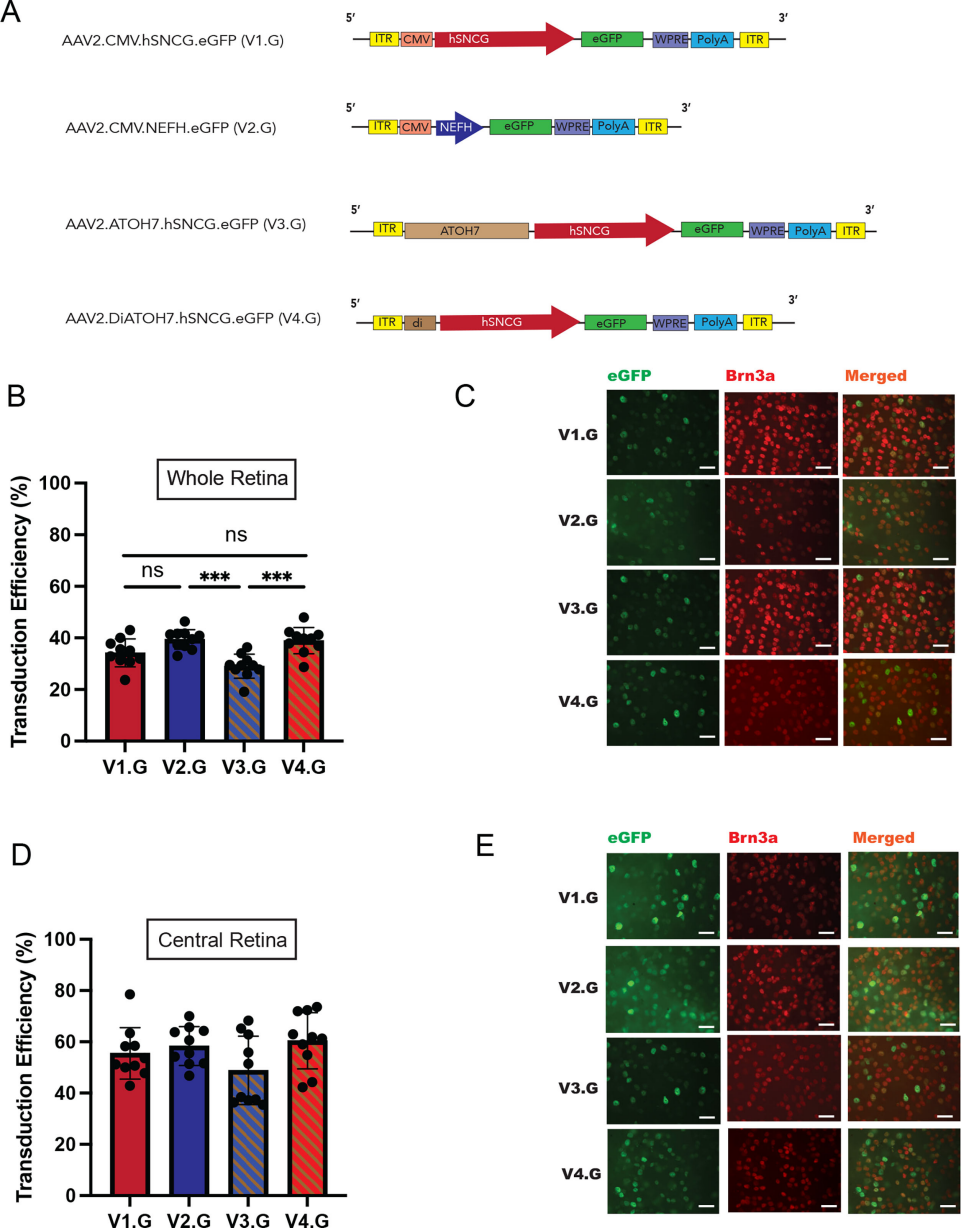
Comparative analysis of novel ATOH7 enhancer–driven and NEFH promoter–driven expression of eGFP. (**A**) Diagram of AAV vector cassettes containing diverse enhancer and promoter sequences to drive eGFP reporter expression.iSV40, SV40 intron; PolyA, poly adenine nucleotide tail; ITR, internal terminal repeat. (**B**) Transduction efficiency at week 2 after intravitreal injection with V1.G, V2.G, V3.G, or V4.G. The percentage of Brn3a^+^ cells (RGCs) that are GFP^+^ in retinal flatmounts are shown. Data are presented as mean ± SEM (*n*  =  10) of the percentage of eGFP^+^ RGCs counted across all retinal regions. (**C**) Representative fundus fluorescence images and Brn3a immunostained flatmount retina of eyes injected with 3 × 10^9^ vg/eye V1.G, V2.G, V3.G, and V4.G vectors. (**D**) Transduction efficiency at week 2 after intravitreal injection with V1.G, V2.G, V3.G, or V4.G. The percentage of Brn3a^+^ cells (RGCs) that are GFP^+^ in retinal flatmounts is shown. Data are presented as mean ± SEM (*n*  =  10) of the percentage of eGFP^+^ RGCs counted across maximally transduced central retinal regions. (**E**) Representative fundus fluorescence images and Brn3a immunostained flatmount retinas of eyes injected with 3 × 10^9^ vg/eye V1.G, V2.G, V3.G, and V4.G vectors in central retina. Values shown are expressed as average ± SEM. The significance of observed differences was determined by one-way ANOVA followed by Tukey's HSD test. ***P < 0.001. Trends toward higher expression that are not marked with an asterisk did not meet statistical significance.

The AAV2 vector has been utilized to optimize the expression of eGFP and other transgenes selectively in RGCs.^8^ We evaluated the efficiency of RGC transduction using the AAV2 vector through intravitreal injection in adult mice. The intravitreal delivery of viral vectors carrying the eGFP transgene under the control of CMV.hSNCG (V1.G), CMV.NEFH (V2.G), ATOH7.hSNCG (V3.G), and diATOH7.hSNCG (V4.G) demonstrated effective delivery with strong expression, devoid of lenticular or retinal compromise or any other adverse effects (Fig. 3). Each vector, at a concentration of 3 × 10^9^ vector genomes (vg), was administered into each experimental eye (*n* = 10). Transduction efficiency was assessed via dual Brn3a immunohistochemical staining and eGFP fluorescence in retinal flatmounts. The quantification of transduction efficiency was performed by determining the percentage of Brn3a^+^ cells that were also eGFP^+^. This quantification was averaged across all retinal areas (Figs. 3B, 3C), as well as in areas of maximal efficiency close to the site of vector delivery in the central retina (Figs. 3D, 3E). Transduction efficiencies between the AAV2.CMV.hSNCG.eGFP (V1.G) and AAV2.CMV.NEFH.eGFP (V2.G) vectors were not statistically different across the entire retina or in the central retina. Similarly, the AAV2.diATOH7.hSNCG.eGFP (V4.G) demonstrated comparable transduction efficiency with the AAV2.CMV.hSNCG.eGFP (V1.G) and AAV2.CMV.NEFH.eGFP (V2.G) vectors. However, the AAV2.ATOH7.hSNCG.eGFP (V3.G) demonstrated a statistically significant reduction in transduction efficiency when quantified across the entire retina. All values were analyzed by one-way ANOVA followed by Tukey's HSD test. The overall infection efficiency of V1.G, V2.G, and V4.G at the clinically acceptable concentration of 3 × 10^9^ vg was 35% in the whole retina (55% in the central retina), 39% in the whole retina (58% in the central retina), and 39% in the whole retina (60% in the central retina), respectively, with the lower V3.G vector measuring 29% in the whole retina (49% in the central retina) at 2 weeks post-injection.

### Comparative Analysis of AAV2 Vector With Optimal Promoter-Driven Expression of the Therapeutic Gene hSIRT1

After finding that the NEFH promoter and DiATOH7 enhancer are capable of driving eGFP expression in RGCs at levels comparable to those of CMV.hSNCG, we next examined whether these enhancer/promoter combinations can also induce similar expression of hSIRT1, which has previously been shown to exert neuroprotective effects in various optic neuropathy models.^9^^–^^11^ The AAV2.CMV.NEFH.SIRT1 (V2.S) is comprised of CMV enhancer (0.304 kb)–NEFH promoter (0.199 kb)–SV40 intron DNA fragment (0.097 kb), along with the poly(A) tail (0.208 kb) and WPRE (0.589 kb), totaling 1.397 kb in length, allowing for a larger therapeutic transgene capacity of 3.303 kb. Additionally, the utilization of the diATOH7 enhancer (0.244 kb)–SNCG promoter (0.953 kb)–SV40 intron DNA fragment (0.097 kb), along with the poly(A) tail (0.208 kb) and WPRE (0.589 kb), although only minimally smaller in size (2.091 kb), with a therapeutic capacity of 2.609 kb, enabled us to evaluate the therapeutic efficacy of the novel ATOH7 sequence with a therapeutic payload, hSIRT1.

**Figure 4.**
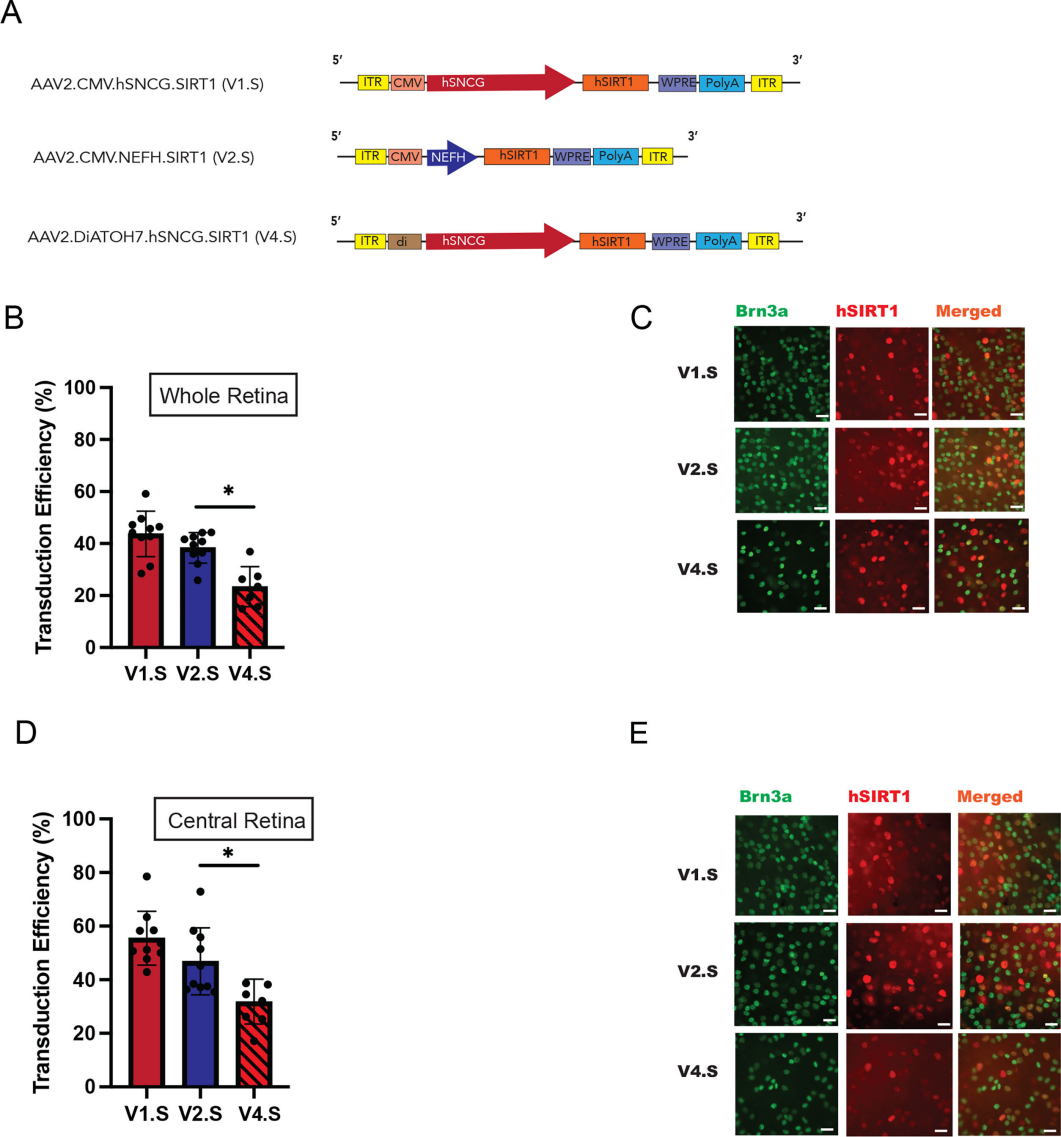
Comparative analysis of DiATOH7 enhancer sequence–driven and NEFH promoter–driven expression of hSIRT1. (**A**) Diagram of AAV vector cassettes containing diverse enhancer and promoter sequences to drive hSIRT1 reporter expression. (**B**) Transduction efficiency involved quantification of the population of SIRT1^+^ RGCs 2 weeks after vector injection. Transduction efficiency was calculated as the percentage of SIRT1^+^Brn3a^+^ cells out of the total number of Brn3a^+^ cells counted in 12 representative fields covering standardized regions across the central, mid-peripheral, and far peripheral retina (*n*  = 10) at week 2 after intravitreal injection with V1.S, V2.S, or V4.S. (**C**) Representative fundus fluorescence images and Brn3a immunostained flatmount retina of eyes injected with 3 × 10^9^ vg/eye V1.S, V2.S, and V4.S vectors. (**D**) Transduction efficiency at week 2 after intravitreal injection with V1.S, V2.S, and V4.S. The percentage of Brn3a^+^ cells (RGCs) that were SIRT1^+^ in the central retina is shown. Data are presented as means ± SEM (*n*  =  10) of the percentage of hSIRT1^+^ RGCs counted across maximally transduced central regions. (**E**) Representative fundus fluorescence images and Brn3a immunostained flatmount retina of eyes injected with 3 × 10^9^ vg/eye V1.S, V2.S, or V4.S vectors in central retina. Values shown are expressed as average ± SEM. *P < 0.05. The significance of observed differences was determined by one-way ANOVA followed by Tukey's HSD test. *P < 0.05. Trends toward higher expression that are not marked with an asterisk did not meet statistical significance.

We generated new AAV vectors (V1.S, V2.S, and V4.S) by replacing the eGFP reporter gene in the V1.G, V2.G, and V4.G vectors with the codon-optimized sequence of the hSIRT1 gene (2.244 kb) (Fig. 4A). Each vector was administered at a dose of 3 × 10^9^ vg/eye by intravitreal injection (*n*  =  10 eyes). After 2 weeks, retinas were collected from all groups and immunostained to detect hSIRT1 protein expression in Brn3a^+^ cells. Our analysis revealed no statistical difference in AAV2-mediated hSIRT1 transduction efficiency in RGCs driven by either the hSNCG or NEFH promoter-CMV enhancer sequence, both in the whole retina (Figs. 4B, 4C) and in the more highly transduced regions of the central retina (Figs. 4D, 4E). However, transduction efficiency was significantly lower when using the diATOH7 enhancer sequence compared with the CMV enhancer sequence.

### AAV2-Mediated RGC-Selective Expression of hSIRT1 Driven by the NEFH Promoter Induces RGC Neuroprotection Similar to the hSNCG Promoter in a Mouse Model of Elevated IOP

**Figure 5.**
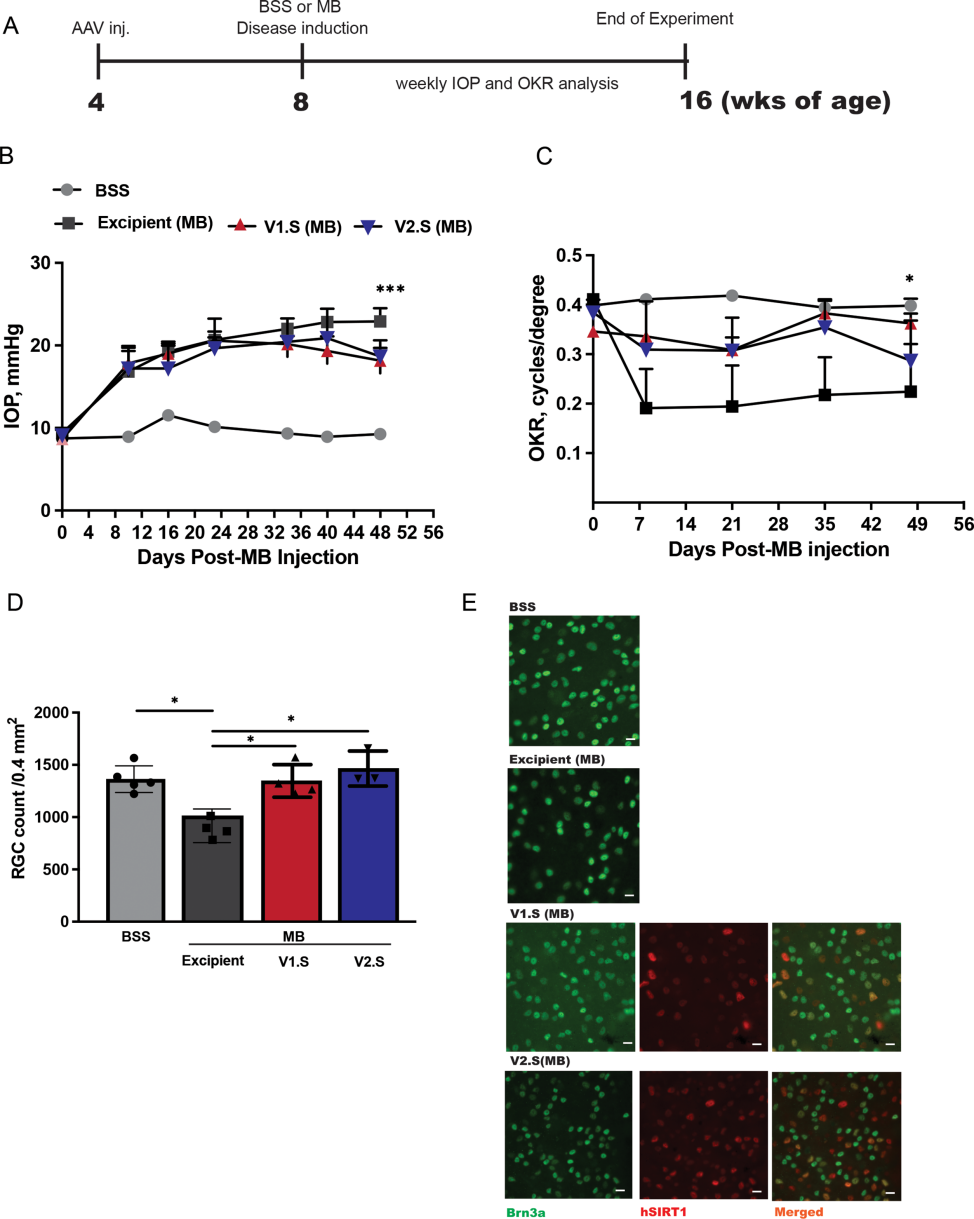
Therapeutic efficacy of NEFH promoter–driven versus hSNCG promoter–driven expression of hSIRT1 in the magnetic MB model of elevated IOP. (**A**) Mice were injected at 4 weeks of age with vectors (V1.S or V2.S) or excipient control, and after an additional 4 weeks the magnetic MB model of ocular hypertension was induced. (**B**) IOP was measured weekly using an iCare tonometer in BSS- (*gray circles*) and MB-injected animals after intravitreal injection of excipient (*black squares*), V1.S (*red triangles*), or V2.S (*blue triangles*) vectors. Mice that received MB injections demonstrated sustained elevated IOP with or without treatment with V1.S or V2.S. (**C**) Visual function was measured weekly by OKRs in BSS- and MB-injected animals after intravitreal injection of excipient (*black squares*), V1.S (*red triangles*), or V2.S (*blue triangles*). Mice that received V1.S or V2.S demonstrated equivalent responses in visual preservation as in control, non–MB-injected eyes, all of which were significantly better than excipient-treated, MB-injected eyes (*P < 0.05). (**D**) RGC survival was measured by counting Brn3a^+^ cells in retinal wholemounts isolated 8 weeks after MB disease induction. MB-injected mice treated with excipient showed significant RGC loss as compared with control BSS injected eyes (*P < 0.05), and eyes treated with V1.S or V2.S also had significantly more RGCs (*P < 0.05) than excipient-treated MB eyes (*n* = 5 eyes/group), as measured by one-way ANOVA followed by Tukey's HSD test. (**E**) Representative wholemount images with Brn3a staining and dual Brn3a (*green*) and hSIRT1 (*red*) staining from vector-treated mice.

The potential neuroprotective effects of the smaller V2.S vector were compared with V1.S in a magnetic microbead (MB) mouse model of ocular hypertension. Both eyes received an injection of 3 × 10^9^ vg/eye of either V1.S or V2.S (*n*  =  5 mice/group) or a sham injection of vector excipient solution (*n*  =  5) 2 weeks before ocular hypertension (Fig. 5A) was induced in the left eyes only. For disease induction, the right eyes of sham-injected mice served as controls and received an intracameral injection of BSS, and the left eyes received magnetic MBs (Fig. 5A). IOP was measured weekly for 7 weeks after intracameral injection of either BSS or MBs, revealing a significant and sustained elevation in IOP in MB-injected eyes compared to BSS-treated eyes. This increase in IOP was evident starting 1 week after injection and persisted throughout the 7-week observation period (Fig. 5B).

The visual function of the treated mice was assessed weekly using OKRs and showed a significant decline in scores in MB-injected eyes compared to BSS-treated eyes (Fig. 5C). Statistical significance was observed between the BSS-treated normotensive group and the excipient-treated MB group only. Mice were euthanized 8 weeks after MB injection, and quantification of RGC numbers (Figs. 5D, 5E) revealed that the significant loss of RGCs induced by MB injection was significantly attenuated by treatment with V1.S and V2.S (excipient, 1002 ± 103 RGCs/mm^2^; AAV2.CMV.hSNCG.SIRT1 (V1.S), 1363 ± 112.8 RGCs/mm^2^; AAV2.CMV.NEFH.SIRT1 (V2.S), 1465 ± 118 RGCs/mm^2^). Immunostaining demonstrated that hSIRT1 expression is maintained in diseased mice (Fig. 5E), similar to retinas from wild-type mice shown in Figure 4.

## Discussion

Using AAV as a vector for delivering therapeutic genes to cells has shown exceptional potential in gene therapy. Still, it also comes with limitations, most notably the limited packaging capacity (4.7 kb). This constraint limits the applicability of AAV in delivering larger therapeutic genes or multiple genes simultaneously. This suggests that, except for increasing the packaging capacity using alternative viral delivery systems, the next solution is to harness more potent and smaller regulatory components in therapeutic constructs. A critical feature of any regulatory components is achieving targeted and specific delivery to the desired cells or tissues. This also points to a need to develop and explore cell-specific components where delivery is not altered; however, the vector can target specific cells that can translate the therapeutic payload.

Our laboratory has also spent considerable effort exploring the therapeutic efficacy of RGC-selective promoters.^8^^–^^11^ This has been largely accomplished using the SNCG promoter; however, the NEFH promoter is also predominantly expressed in mature neurons, making it an attractive candidate for driving gene expression selectively in these cells.^19^ In this study, we engineered vectors to compare the effectiveness of the SNCG promoter with the smaller NEFH promoter. Although NEFH proved as strong as the SNCG promoter, it did not exhibit more robust expression, transduction efficiency, or therapeutic efficacy, but, importantly, these findings were equivalent. Thus, this finding is significant, as NEFH presents several characteristics that make it a potentially preferred choice for vector design. NEFH has demonstrated excellent RGC specificity through intravitreal injection and eGFP expression analysis,^19^ and it boasts an overall smaller promoter sequence compared to SNCG. Together, these properties and the current findings support the design of future studies using this construct to explore the potential role of large candidate therapeutic genes, such as those that have shown promise in the literature including neurotrophins^30^^–^^32^ or genes to enhance NAD metabolism.^33^^,^^34^

ATOH7 sequences have also emerged as valuable enhancers in gene regulation, particularly in neurogenesis and retinal development.^22^^,^^35^ Its regulatory sequences act as enhancers that promote the expression of key genes involved in retinal development,^22^ providing exquisite regulation of downstream genes with precise temporal and spatial expression patterns in developing retina and retinal organoids. Leveraging previously published data identifying smaller sequences responsible for robust transcription, we selected these sequences for our studies to analyze transduction efficiency. However, hSIRT1 expression was not as efficient with the DiATOH7 enhancer as with CMV.

Overall, using hSIRT1 expression under regulation of the CMV enhancer and either the SNCG or NEFH promoter results demonstrated these two constructs mediate equivalent neuroprotective effects on RGC number and function. Our results therefore support the use of either construct in the design of novel RGC gene therapies, with the potential to examine effects of larger therapeutic genes using the NEFH promoter in future studies.

## References

[1] He X, Fu Y, Ma L, et al. AAV for gene therapy in ocular diseases: progress and prospects. *Research (Wash D C)*. 2023; 6: 0291. 38188726 10.34133/research.0291 PMC10768554

[2] Dunbar CE, High KA, Joung JK, Kohn DB, Ozawa K, Sadelain M. Gene therapy comes of age. *Science*. 2018; 359(6372): eaan4672. 29326244 10.1126/science.aan4672

[3] Lundstrom K. Viral vectors in gene therapy. *Diseases*. 2018; 6(2): 42. 29883422 10.3390/diseases6020042 PMC6023384

[4] Balakrishnan B, Jayandharan GR. Basic biology of adeno-associated virus (AAV) vectors used in gene therapy. *Curr Gene Ther*. 2014; 14(2): 86–100. 24588706 10.2174/1566523214666140302193709

[5] Fischer MD, Ochakovski GA, Beier B, et al. Changes in retinal sensitivity after gene therapy in choroideremia. *Retina*. 2020; 40(1): 160–168. 30308560 10.1097/IAE.0000000000002360

[6] Wang D, Tai PWL, Gao G. Adeno-associated virus vector as a platform for gene therapy delivery. *Nat Rev Drug Discov*. 2019; 18(5): 358–378. 30710128 10.1038/s41573-019-0012-9 PMC6927556

[7] Nieuwenhuis B, Laperrousaz E, Tribble JR, et al. Improving adeno-associated viral (AAV) vector-mediated transgene expression in retinal ganglion cells: comparison of five promoters. *Gene Ther*. 2023; 30(6): 503–519. 36635457 10.1038/s41434-022-00380-z PMC10284706

[8] Chaqour B, Duong TT, Yue J, et al. AAV2 vector optimization for retinal ganglion cell-targeted delivery of therapeutic genes. *Gene Ther*. 2024; 31(3-4): 175–186. 38200264 10.1038/s41434-023-00436-8

[9] Ross AG, Chaqour B, McDougald DS, et al. Selective upregulation of SIRT1 expression in retinal ganglion cells by AAV-mediated gene delivery increases neuronal cell survival and alleviates axon demyelination associated with optic neuritis. *Biomolecules*. 2022; 12(6): 830. 35740955 10.3390/biom12060830 PMC9221096

[10] Ross AG, McDougald DS, Khan RS, et al. Rescue of retinal ganglion cells in optic nerve injury using cell-selective AAV mediated delivery of SIRT1. *Gene Ther*. 2021; 28(5): 256–264. 33589779 10.1038/s41434-021-00219-z PMC8149296

[11] Yue J, Khan RS, Duong TT, et al. Cell-specific expression of human SIRT1 by gene therapy reduces retinal ganglion cell loss induced by elevated intraocular pressure. *Neurotherapeutics*. 2023; 20(3): 896–907. 36941497 10.1007/s13311-023-01364-6 PMC10275821

[12] Buch PK, Bainbridge JW, Ali RR. AAV-mediated gene therapy for retinal disorders: from mouse to man. *Gene Ther*. 2008; 15(11): 849–857. 18418417 10.1038/gt.2008.66

[13] Hermonat PL, Quirk JG, Bishop BM, Han L. The packaging capacity of adeno-associated virus (AAV) and the potential for wild-type-plus AAV gene therapy vectors. *FEBS Lett*. 1997; 407(1): 78–84. 9141485 10.1016/s0014-5793(97)00311-6

[14] Cen LP, Park KK, So KF. Optic nerve diseases and regeneration: how far are we from the promised land? *Clin Exp Ophthalmol*. 2023; 51(6): 627–641. 37317890 10.1111/ceo.14259 PMC10519420

[15] Levin LA, Patrick C, Choudry NB, Sharif NA, Goldberg JL. Neuroprotection in neurodegenerations of the brain and eye: lessons from the past and directions for the future. *Front Neurol*. 2022; 13: 964197. 36034312 10.3389/fneur.2022.964197 PMC9412944

[16] Khan RS, Ross AG, Aravand P, Dine K, Selzer EB, Shindler KS. RGC and vision loss from traumatic optic neuropathy induced by repetitive closed head trauma is dependent on timing and force of impact. *Transl Vis Sci Technol*. 2021; 10(1): 8. 10.1167/tvst.10.1.8 PMC7794277 33505775

[17] Shindler RE, Yue J, Chaqour B, Shindler KS, Ross AG. Repeat Brn3a immunolabeling rescues faded staining and improves detection of retinal ganglion cells. *Exp Eye Res*. 2023; 226: 109310. 36400286 10.1016/j.exer.2022.109310 PMC9839618

[18] Khan RS, Fonseca-Kelly Z, Callinan C, Zuo L, Sachdeva MM, Shindler KS. SIRT1 activating compounds reduce oxidative stress and prevent cell death in neuronal cells. *Front Cell Neurosci*. 2012; 6: 63. 23293585 10.3389/fncel.2012.00063 PMC3533205

[19] Millington-Ward S, Chadderton N, Berkeley M, et al. Novel 199 base pair NEFH promoter drives expression in retinal ganglion cells. *Sci Rep*. 2020; 10(1): 16515. 33020509 10.1038/s41598-020-73257-z PMC7536420

[20] Chaffiol A, Caplette R, Jaillard C, et al. A new promoter allows optogenetic vision restoration with enhanced sensitivity in macaque retina. *Mol Ther*. 2017; 25(11): 2546–2560. 28807567 10.1016/j.ymthe.2017.07.011 PMC5675708

[21] Wu F, Bard JE, Kann J, et al. Single cell transcriptomics reveals lineage trajectory of retinal ganglion cells in wild-type and Atoh7-null retinas. *Nat Commun*. 2021; 12(1): 1465. 33674582 10.1038/s41467-021-21704-4 PMC7935890

[22] Miesfeld JB, Ghiasvand NM, Marsh-Armstrong B, et al. The Atoh7 remote enhancer provides transcriptional robustness during retinal ganglion cell development. *Proc Natl Acad Sci USA*. 2020; 117(35): 21690–21700. 32817515 10.1073/pnas.2006888117 PMC7474671

[23] Bennicelli J, Wright JF, Komaromy A, et al. Reversal of blindness in animal models of leber congenital amaurosis using optimized AAV2-mediated gene transfer. *Mol Ther*. 2008; 16(3): 458–465. 18209734 10.1038/sj.mt.6300389 PMC2842085

[24] Van Vliet KM, Blouin V, Brument N, Agbandje-McKenna M, Snyder RO. The role of the adeno-associated virus capsid in gene transfer. *Methods Mol Biol*. 2008; 437: 51–91. 18369962 10.1007/978-1-59745-210-6_2 PMC7120696

[25] Quinn TA, Dutt M, Shindler KS. Optic neuritis and retinal ganglion cell loss in a chronic murine model of multiple sclerosis. *Front Neurol*. 2011; 2: 50. 21852980 10.3389/fneur.2011.00050 PMC3151613

[26] Prusky GT, Alam NM, Beekman S, Douglas RM. Rapid quantification of adult and developing mouse spatial vision using a virtual optomotor system. *Invest Ophthalmol Vis Sci*. 2004; 45(12): 4611–4616. 15557474 10.1167/iovs.04-0541

[27] Prusky GT, Alam NM, Douglas RM. Enhancement of vision by monocular deprivation in adult mice. *J Neurosci*. 2006; 26(45): 11554–11561. 17093076 10.1523/JNEUROSCI.3396-06.2006 PMC6674777

[28] Khan RS, Baumann B, Dine K, et al. Dexras1 deletion and iron chelation promote neuroprotection in experimental optic neuritis. *Sci Rep*. 2019; 9(1): 11664. 31406150 10.1038/s41598-019-48087-3 PMC6690882

[29] Nadal-Nicolas FM, Galindo-Romero C, Lucas-Ruiz F, et al. Pan-retinal ganglion cell markers in mice, rats, and rhesus macaques. *Zool Res*. 2023; 44(1): 226–248. 36594396 10.24272/j.issn.2095-8137.2022.308 PMC9841181

[30] Tsai JC. Innovative IOP-independent neuroprotection and neuroregeneration strategies in the pipeline for glaucoma. *J Ophthalmol*. 2020; 2020: 9329310. 33014446 10.1155/2020/9329310 PMC7512103

[31] Ji JZ, Elyaman W, Yip HK, et al. CNTF promotes survival of retinal ganglion cells after induction of ocular hypertension in rats: the possible involvement of STAT3 pathway. *Eur J Neurosci*. 2004; 19(2): 265–272. 14725620 10.1111/j.0953-816x.2003.03107.x

[32] Chitranshi N, Dheer Y, Abbasi M, You Y, Graham SL, Gupta V. Glaucoma pathogenesis and neurotrophins: focus on the molecular and genetic basis for therapeutic prospects. *Curr Neuropharmacol*. 2018; 16(7): 1018–1035. 29676228 10.2174/1570159X16666180419121247 PMC6120108

[33] Sporny M, Guez-Haddad J, Khazma T, et al. Structural basis for SARM1 inhibition and activation under energetic stress. *eLife*. 2020; 9: e62021. 33185189 10.7554/eLife.62021 PMC7688312

[34] Bosanac T, Hughes RO, Engber T, et al. Pharmacological SARM1 inhibition protects axon structure and function in paclitaxel-induced peripheral neuropathy. *Brain*. 2021; 144(10): 3226–3238. 33964142 10.1093/brain/awab184 PMC8634121

[35] Song WT, Zhang XY, Xia XB. Atoh7 promotes the differentiation of retinal stem cells derived from Müller cells into retinal ganglion cells by inhibiting Notch signaling. *Stem Cell Res Ther*. 2013; 4(4): 94. 23945288 10.1186/scrt305 PMC3854761

